# Clinical Use of Cisplatin Liposomes for Patients With Refractory Advanced Cancer

**DOI:** 10.7759/cureus.73181

**Published:** 2024-11-06

**Authors:** Yasuo Komura, Shintarou Kimura, Tomoko Katagiri, Yuumi Hirasawa, Hiromi Muranishi, Koichiro Homma

**Affiliations:** 1 Internal Medicine, Rinku Medical Clinic Advanced Medical Center, Osaka, JPN; 2 Environmental Science, StateArt, Inc., Tokyo, JPN; 3 Research and Development, StateArt, Inc., Tokyo, JPN; 4 Medical Technology, StateArt, Inc., Tokyo, JPN; 5 Internal Medicine, Kyotooike Medical Clinic, Kyoto, JPN; 6 Emergency and Critical Care Medicine, Keio University School of Medicine, Tokyo, JPN

**Keywords:** adverse drug reaction, circulating tumor cells, cisplatin liposomes, enhanced permeability and retention effect, nephrotoxicity, pulmonary metastasis of pancreatic cancer, quality of life, recurrent liver metastases from primary nasal cancer

## Abstract

This study aimed to evaluate the safety and efficacy of cisplatin (CDDP) liposomes. A patient with multiple recurrent liver metastases from metastatic nasal carcinoma was administered CDDP liposomes with consent. Magnetic resonance imaging showed that the patient remained stable disease; however, no apparent side effects were observed, and blood draw data showed no worsening of renal function. Patients undergoing partial pancreatectomy and jejunoileal biliary anastomosis for biliary tract cancer who consented to receive CDDP liposomes demonstrated a partial response on angiographic computed tomography; however, they showed slight fatigue. To our knowledge, the present study is the first in Japan to suggest that liposomalization of CDDP may have anticancer effects while alleviating renal damage and bone marrow suppression.

## Introduction

Cisplatin (CDDP) has been widely used as a first-line therapy for malignant tumors owing to its potent antitumor effect since its discovery by Rosenberg et al. in 1965 [1]. CDDP incorporated into cancer cells preferentially binds to the nitrogen atom at the 7-position of purine bases, especially guanine bases in the nucleus, due to a water molecule's substitution of chloride ligands, bridging two adjacent purine bases [2]. The various DNA-platinum covalent adducts that are formed inhibit transcription factors and polymerases and cause chromatin disruption, ultimately inducing apoptosis in cancer cells [3]. CDDP-based chemotherapy is a central component of several curative approaches for patients with malignant diseases, including gastric, esophageal, lung, ovarian, testicular, and head and neck cancers [4-9]. CDDP has a high tumor regression effect; however, it causes serious side effects and severely impairs the patient's quality of life (QOL) [10]. Gastrointestinal symptoms such as nausea, vomiting, and loss of appetite, which occur in many patients, are particularly severe in patients receiving various anticancer agents and are frequently treated using antiemetic agents [11]. The most severe side effects are kidney failure and other kidney dysfunctions [11,12]. Infusing patients with large volumes of fluids and using diuretics to increase urine output and reduce nephrotoxicity is mandatory for these side effects. However, these measures can severely interfere with a patient's QOL. In addition, there is concern that CDDP may cause neurotoxicity and a decrease in leucocytes, leading to a decline in immune function due to myelosuppression [12]. Therefore, it is essential to fully monitor the patient's condition while conducting each function’s tests to ensure safety and QOL when treating with CDDP. Furthermore, several researchers have reported that CDDP causes adverse side effects due to its small molecular weight and indiscriminate distribution in normal tissues [13-16]. These reports are based on the following theory: polymerization of CDDP into polymeric polymers can accumulate CDDP in tumor tissues through the enhanced permeability and retention (EPR) effect and mitigate adverse drug reactions by reducing its distribution in normal tissues.

We have previously reported that a complex of styrene-co-maleic acid (SMA) encapsulated CDDP (SMA-CDDP) accumulates in tumors through the EPR effect and can suppress cancer without harmful side effects in mice transplanted with cancer cells [16]. However, SMA-CDDP has the disadvantage of undergoing several days of chemical synthesis [15], making it less convenient for clinical use. Our research team previously reported a clinical case in which indocyanine green (ICG) encapsulated liposomes (ICG liposomes) were accumulated in a tumor and demonstrated therapeutic efficacy with photodynamic therapy [17]. Therefore, we used liposomes in the present study because of guaranteed tumor accumulation and safety in clinical practice owing to the EPR effect. They are easily prepared and suitable for encapsulating low molecular weight compounds, such as CDDP.

The present study reported a clinical case in which CDDP liposomes were administered to two patients, one with multiple recurrent liver metastases from metastatic nasal cancer and the other who underwent partial pancreatectomy and jejunal biliary anastomosis for biliary tract cancer, to evaluate their safety and efficacy.

## Case presentation

Ethical review and informed consent

This study was approved by the Ethics Review Committee at the IGT Clinic on December 21, 2022, under approval number 19. Participants provided written informed consent before the clinical trial. This study was conducted in accordance with the Declaration of Helsinki and the Ethical Guidelines for Medical Research Involving Human Subjects established by the Japanese Ministry of Health, Labor, and Welfare.

General disability and administration site conditions were diagnosed by the physician according to Common Terminology Criteria for Adverse Events (CTCAE) v5.0 - Japan Clinical Oncology Group (JCOG) before and after administering the CDDP liposome treatment.

Methods

Preparation of CDDP Liposomes

**Figure 1:**
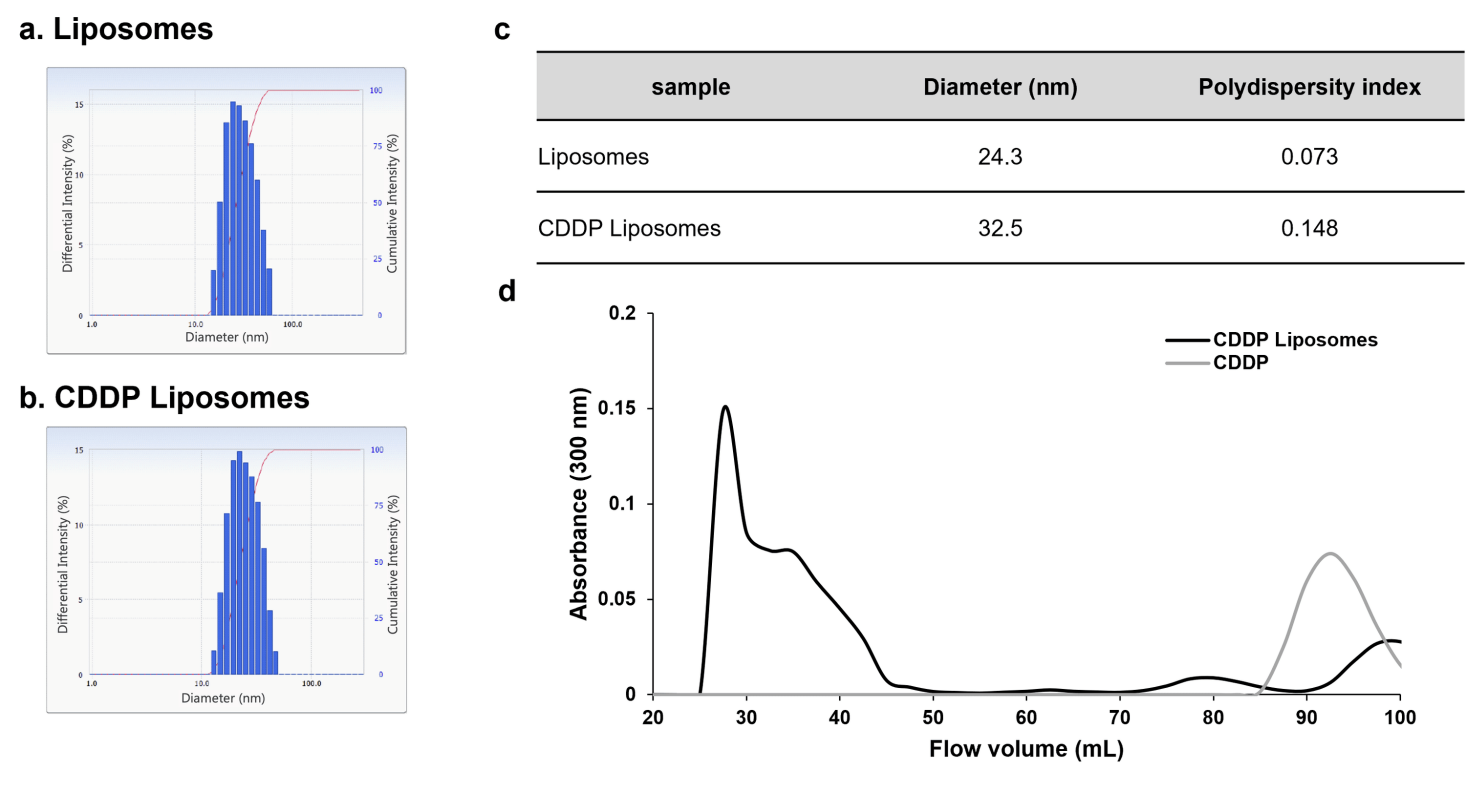
(a-c) Physiochemical characteristics of CDDP liposomes compared with liposomes. The size of liposomes is adjusted to ≤100 nm, which is optimal for EPR effects. CDDP liposomes show a similar particle size distribution as before encapsulation, although slightly larger. (d) Comparison of CDDP and CDDP liposomes using gel filtration chromatography. CDDP liposomes were detected in the 25-50 mL fraction, whereas CDDP alone was detected in the 85-100 mL fraction. CDDP, cisplatin; EPR, enhanced permeability and retention

1,2-Dimyristoyl-sn-glycero-3-phosphocholine (DMPC) was purchased from Yuka-Sangyo Co., Ltd. (Tokyo, Japan), and CDDP was purchased from Nichi-Iko (Sogawa, Japan). Liposomes were prepared as previously reported [17]. Briefly, DMPC was dissolved in a 5% glucose solution at a concentration of 8.85 mM using a Bransonic® CPX8800H-J Ultrasonic Cleaner (Branson Ultrasonic Co., Ltd., Danbury, CT) and then sonicated at 40 kHz for 60 min under 45 °C. Subsequently, the liposomes were purified using sterile filtration through a 0.20 μm pore size filter. The particle size of liposomes was determined using an ELSZ-2000 (Otsuka Electronics Co., Ltd., Osaka, Japan) (Figures 1a-1c). CDDP liposomes were prepared by mixing 10 mg/20 mL CDDP and 8.85 mM/10 mL liposomes and sterile-filtered through a 0.20 mm filter. Liposomalization of CDDP was performed with gel filtration chromatography using a Sephadex® G-25 column (Merck, German; Figure 1d).

CDDP Liposome Therapeutic Intervention

CDDP (10 mg) was mixed with 300 mg of DMPC-derived liposomes, purified using a 0.2 μm filter, diluted to 100 mL with saline (Hikari Pharmaceutical Co., Ltd., Tokyo, Japan), and then injected intravenously at 2 mL/kg. Immediately after CDDP administration, 2 g/20 mL sodium thiosulfate (Nichi-Iko Pharmaceutical Co., Ltd., Tokyo, Japan) was administered intravenously.

Examination of Circulating Tumor Cells

Patients had their blood drawn on their first visit for circulating tumor cells (CTC) testing. Obtained blood samples were immediately transported to Research Genetics Cancer Center International (RGCC; GmBH, Switzerland) for analysis. We received the test report.

Case 1: Administration of CDDP liposomes to a patient with recurrence of multiple liver metastases from metastatic nasal cancer

A 58-year-old man with a height of 165 cm, weight of 60 kg, and performance status (PS) of 0 gave informed consent to participate in our clinical trial. His medical history is as follows. He had a recurrence of multiple liver metastases in 2019, despite a complete response to selective administration of CDDP and radiotherapy for stage-1 nasal cancer in 2012. He demonstrated a partial response after 12 courses of systemic chemotherapy with CDDP and irinotecan for the recurrence of multiple liver metastases. However, during this therapy, he experienced nausea, malaise, and lingering side effects, such as numbness in his fingers, and his QOL was greatly reduced. He was seen at our hospital in October 2020 while receiving standard treatment at another hospital.

**Figure 2:**
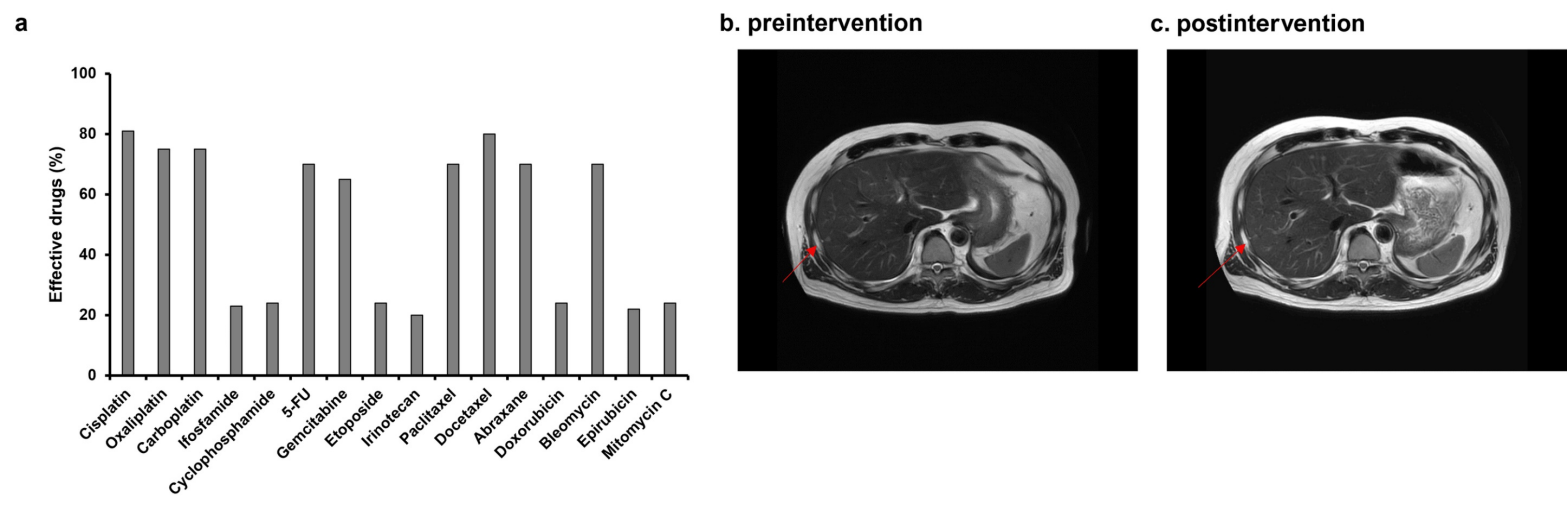
(a) The sensitivity of various anticancer drugs to patient-derived cancer cells was examined using CTC testing, and the results showed that CDDP had the most antitumor effect. (b) Magnetic resonance imaging (MRI) T2WI analysis of the liver of a 58-year-old man with recurrent liver metastases from primary nasal cancer on October 4, 2022. (c) The patient underwent an MRI T2WI analysis again on January 4, 2023. An invariant tumor size of liver metastases is observed on MR images. CDDP, cisplatin; CTC, circulating tumor cells

Gene therapy using p53 and TRAIL lentivectors combined with intravenous infusion of high-dose ascorbic acid resulted in almost complete remission. For approximately one year, we tried to prevent cancer recurrence in the patient by administering high-dose ascorbic acid; however, two recurrent lesions were observed in the patient’s liver in October 2022, and he was recommended systemic chemotherapy with CDDP and irinotecan as standard treatment at another hospital. The patient refused because he was concerned that his QOL would deteriorate tremendously like before. Based on previously successful results of combined therapy with CDDP and irinotecan and the favorable response to CDDP in the CTC test (Figure 2a), we planned therapeutic intervention with CDDP liposomes. We added 100 mL of saline to 30 mL of CDDP liposomes and administered this treatment to the patients once weekly from October 22 to December 20, 2022. After the intervention, the patient only complained of mild fatigue (Table 1), and no other adverse side effects were observed (Tables 1-2). The intervention was completed without any evidence of exacerbation of renal function in blood test data. Subsequently, magnetic resonance imaging (MRI) examination showed stable disease (SD) (Figures 2b, 2c).

**Table 1:** Physician toxicity evaluation according to the Common Terminology Criteria for Adverse Events (CTCAE) v5.0 - Japan Clinical Oncology Group (JCOG) after CDDP liposome treatment intervention. CDDP, cisplatin.

Characteristics	Pre-intervention	Post-intervention	Pre-intervention	Post-intervention
Age (years)	57		52	
Sex	Male		Female	
Height	165.0		160.0	
Weight	60.0		57.0	
PS	0		0	
Chills	0	0	0	0
Edema face	0	0	0	0
Fatigue	0	1	0	1
Gait disturbance	0	0	0	0
Infusion site extravasation	0	0	0	0
Injection site reaction	0	0	0	0
Malaise	0	0	0	0
Pain	0	0	0	0

**Table 2:** Clinical characteristics of a 58-year-old patient who received CDDP liposomes treatment. TP, total protein; ALB, albumin; T-Bil, total bilirubin; ALP, alanine phosphotransferase; AST, aspartate aminotransferase; ALT, alanine aminotransferase; LD, lactate dehydrogenase; γ-GT, gamma-glutamyl transferase; Ch-E, cholinesterase; GLU, glucose; HbA1c, hemoglobin A1C; TC, total cholesterol; TG, triglyceride; UN, urea nitrogen; CRE, creatinine; eGFR, estimated glomerular filtration rate; CRP, C-reactive protein; WBC, white blood cell; RBC, red blood cell; Hgb, hemoglobin; Hct, hematocrit; MCV, mean corpuscular volume; MCHC, mean corpuscular hemoglobin concentration; PLT, platelet; MYELO, myelocyte; MET. M, Metamyelocyte; NEUT, neutrophil; EOS, eosinophil; BASO, basophil; LYMP, lymphocyte. AT. LY, atypical lymphocyte; Mono, monocyte; CDDP, cisplatin

Test items	Reference ranges	Unit	12/6/22	12/13/22	12/20/22	12/27/22	1/6/23	1/11/23	1/16/23	1/24/23	1/31/23
TP	6.5–8.2	g/dL	7.4	7.5	7.4	7.7	7.0	7.3	7.5	7.3	7.8
ALB	3.8–5.2	g/dL	4.5	4.5	4.7	4.9	4.7	4.5	4.8	4.6	4.7
T-Bil	0.2–1.2	mg/dL	0.5	0.5	0.4	0.6	0.4	0.4	0.6	0.4	0.5
ALP	38–113	U/L	39	38	41	36	32	33	35	31	46
AST	10–40	U/L	17	25	17	18	16	16	19	18	22
ALT	6–40	U/L	16	17	14	14	13	13	13	12	17
LD	124–222	U/L	127	219	139	133	125	114	134	134	154
γ-GT	80 ≦	U/L	38	43	42	40	34	36	40	35	46
Ch-E	200–465	U/L	286	268	289	301	277	270	291	265	281
GLU	70–109	mg/dL	116	122	144	114	160	165	136	147	149
TC	130–219	mg/dL	226	205	210	215	206	213	220	192	214
TG	35–149	mg/dL	185	126	145	140	148	191	102	110	177
UN	8.0–21.0	mg/dL	11.9	13.9	16.7	14.3	14.9	17.3	15.8	18.4	17.4
CRE	0.60–1.15	mg/dL	0.91	1.05	1.05	1.01	1.03	1.02	1.08	1.05	1.01
eGFR	1.73 m^2^ ≧ 60	mL/min	67.4	57.6	57.6	60.1	59.5	59.5	55.9	57.6	59.8
CRP	0.30 ≦	mg/dL	0.17	1.07	0.37	0.13	0.27	0.86	0.76	0.71	1.75
WBC	38–98	10^2^/μL	45	33	48	45	41	56	42	43	47
RBC	420–570	10^4^/μL	423	427	422	427	413	411	473	408	421
Hgb	13.2–17.6	g/dL	12.7	12.8	12.7	13.0	12.4	12.1	13.1	12.4	12.7
Hct	39.2–51.8	%	40.4	40.9	41.1	40.4	40.0	39.6	41	38.9	41.3
MCV	83.0–101.5	fL	95.5	95.8	97.4	94.6	96.9	96.4	93.8	95.3	98.1
MCH	28.0–34.5	pg	30.0	30.0	30.1	30.4	30.0	29.4	30.0	30.4	30.2
MCHC	31.5–35.5	％	31.4	31.3	30.9	32.2	31.0	30.6	32.0	31.9	30.8
PLT	14.0–36.0	10^4^/μL	23.7	25.0	27.4	27.2	27.3	27.1	29.9	27.5	29.4
MYELO	0.0	%	0.0	0.0	0.0	0.0	0.0	0.0	0.0	0.0	0.0
MET. M	0.0	%	0.0	0.0	0.0	0.0	0.0	0.0	0.0	0.0	0.0
NEUT	36.0–69.0	%	71.4	59.5	67.7	70.4	66.5	73.4	68.5	69.7	71.9
EOS	1.0–5.0	%	1.3	3.1	1.5	0.9	1.5	0.9	1.4	0.9	1.1
BASO	0.0–2.0	%	1.1	1.8	1.3	1.1	0.5	0.5	0.7	0.9	0.8
LYMP	27.0–53.0	%	20.4	27.0	22.8	22.5	24.7	19	23.4	22.2	20.7
AT. LY	0.0	%	0.0	0.0	0.0	0.0	0.0	0.0	0.0	0.0	0.0
MONO	2.0–10.0	%	5.8	8.6	6.7	5.1	6.8	6.2	6.0	6.3	5.5

Case 2: Administration of CDDP liposomes to a patient undergoing partial pancreatic resection and jejunoileal anastomosis for biliary tract cancer

**Figure 3:**
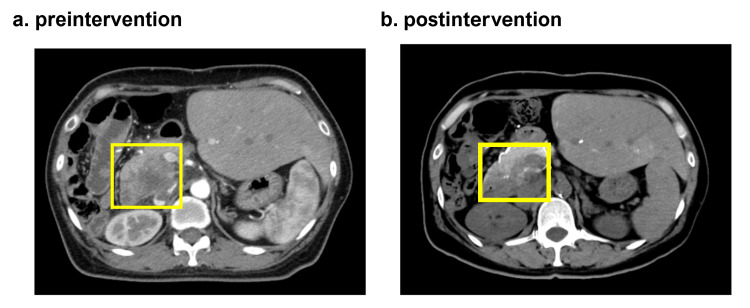
(a) Contrast computed tomography (CT) image of a woman with postoperative cholangiocarcinoma and pulmonary metastasis of pancreatic cancer taken before CDDP therapeutic intervention. A mass was observed in her pancreatic head after partial hepatic right lobe resection. (b) Her CT angiography image taken with contrast from the arteria hepatica propria one week after the preintervention image (a) was taken. Her pancreatic head tumor was observed to have shrunk. CDDP, cisplatin

A 52-year-old woman, with a height of 160 cm, weight of 57 kg, and PS of 0, provided informed consent to participate in our clinical trial. Her past medical history is as follows. In October 2021, she was diagnosed with well-differentiated adenocarcinoma by biopsy after a computed tomography (CT) and autopsy ultrasonography (AUS) scan revealed masses in her pancreas and left lung. Although she underwent micro-RNA capsule gene therapy at another hospital in May and October 2022, she visited our hospital in January 2023 due to progressive disease (PD). She agreed to our proposal of an integrated approach using CDDP liposomes and commenced treatment. On January 16, 100 mL of saline was added to a standard formulation of CDDP liposomes (CDDP 10 mg + liposome 300 mg) and administered to her; as a result, she experienced mild fatigue on the day (Table 1). Therefore, two days later, on January 18, we reduced the CDDP formula to 3.3 mg while keeping liposome content at 300 mg and added 100 mL of normal saline, which was administered to her via an intravenous drip. Our attempts were successful, and she did not experience any adverse side effects from the CDDP liposomes. Subsequently, we applied hyperthermia to her on January 20, and then, an angiographic CT scan diagnosed her with a partial response (PR) on January 23 (Figure 3).

## Discussion

Despite the increase in new anticancer agents, such as molecularly targeted drugs and immune checkpoint inhibitors [18,19], CDDP is still considered the first treatment choice for many patients with cancer in clinical practice. This is because it has a high antitumor effect against various cancers, including gastric, esophageal, lung, ovarian, testicular, and head and neck cancers [4-9], and potential synergistic effects when combined with other types of anticancer agents and radiation therapy [20,21]. Our research group previously reported that CDDP combined with a Lentiviral vector with tumor suppressor genes, including p53 and p16, and phosphatase and tensin homolog (PTEN)-mediated gene therapy, inhibited the growth of non-muscle invasive bladder cancer (NMIBC) [22]. Despite CDDP’s usefulness and versatility, its use is often discouraged based on physician judgment or rejected by patients because of the occurrence of gastrointestinal symptoms, such as chemotherapy-induced nausea and vomiting (CINV) and, in severe cases, neurological disorders, immunosuppression, and renal toxicity [10-12]. Aprepitant, 5-HT3 receptor antagonists, and dexamethasone are used to counter the side effects of CDDP, primarily to prevent CINV. Aprepitant and 5-HT3 receptor antagonists are metabolized by hepatic cytochrome P-450s such as CYP3A4, and therefore demonstrate a dose-dependent inhibitory effect on CYP3A4 [23], and may interact with concomitant drugs, including antineoplastic agents, which may cause excessive increases in blood levels because they are metabolized primarily in the liver, thus limiting their use in patients with severe liver impairment. Steroidal agents, such as dexamethasone, have been shown to prevent allergies and antiemetics; however, there is a concern regarding their immunosuppressive effects [24]. Because higher-grade tumors require higher doses of CDDP, such patients require higher doses of antiemetics. Therefore, physicians and patients must be more concerned about the side effects of CINV prophylaxis drugs and CDDP. Furthermore, the development of anticancer drugs that accumulate only in tumors without affecting other organs has been eagerly awaited.

With the development of drug delivery in recent years, polymerized anticancer drugs based on micelles and liposomes have been attracting attention as a countermeasure for serious side effects such as CDDP-mediated nephrotoxicity [13-16]. Clinical trials have also been conducted on CDDP modified with polyethylene glycol (PEG) and liposomes; however, these have not yet been used in actual clinical practice in Japan. The present study is the first in Japan to reveal that liposomalized CDDP may alleviate CDDP-mediated adverse drug reactions.

In the present study, we conducted a clinical trial on CDDP liposomes and combined sodium thiosulfate with CDDP liposomes to minimize CDDP-mediated adverse drug reactions. Even if the liposome formulation collapses before incorporation into cancer cells and CDDP leaks into the bloodstream, a low incidence of adverse effects due to toxicity in normal cells is expected because sodium thiosulfate chelates CDDP.

Neither of the participants in the present study showed abnormal increases in blood tests for clinical markers of nephrotoxicity, such as urea nitrogen (UN) or creatinine, after the CDDP liposome treatment intervention. In addition, these participants showed no abnormalities in their blood leukocytes, suggesting that liposomalization of CDDP may also reduce myelosuppression. The 58-year-old male patient was diagnosed with a metastatic liver tumor that continued to grow; however, some therapeutic benefits were observed because his tumor remained stable disease during the administration period of the CDDP liposome therapeutic intervention. The 52-year-old female patient showed mild fatigue during treatment with 10 mg encapsulated CDDP liposomes. The patient underwent contrast CT shortly after she arrived in Japan, revealing that the mild fatigue may be due to dehydration. This is because, during transcatheter arterial chemoembolization (TACE), approximately 1,500 mg of infusion fluid and 0.75 mg of Palonosetron hydrochloride were used as anti-nausea drugs, and 10 mg of undiluted CDDP was used, without the occurrence of nausea. Therefore, we thought that reducing the CDDP dose from 10 to 3.3 mg and liposomalizing it would be sufficient to treat patients without the occurrence of nausea. We observed that CDDP liposomes were safe and achieved tumor regression for the 58-year-old participant.

**Figure 4:**
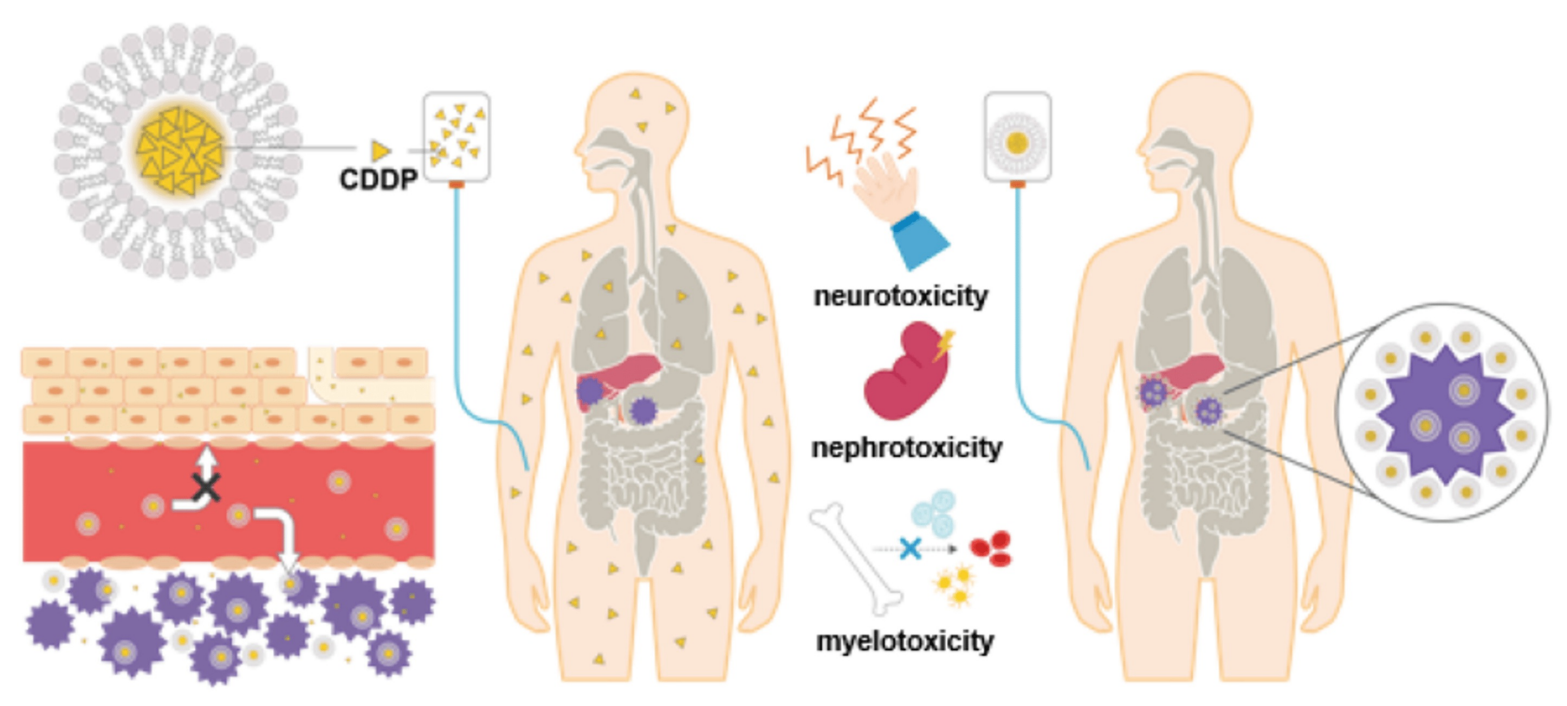
Schematic diagram showing the metabolic pathways of CDDP and CDDP liposomes. CDDP, cisplatin

CDDP liposome intervention demonstrated some antitumor efficacy in both participants in the present study. The effect of sodium thiosulfate cannot be ruled out in terms of the side effects caused by CDDP in the present study [25]; however, we believe that the present results are due to the liposomalization of CDDP, as shown in Figure 4, and previous studies that used similar liposomes have also reported liposome-related EPR effects [17]. Furthermore, previous reports of CDDP liposomes reducing cytotoxicity in humans in clinical trials and in vitro studies support the present study’s results [26,27].

## Conclusions

To the best of our knowledge, the present study is the first in Japan to reveal that liposomalized CDDP may alleviate CINV and CDDP-mediated adverse drug reactions, such as renal damage and immunosuppression. In the future, we will increase the number of participants who consent to participate in a study to test the safety and efficacy of CDDP liposomes in treating various symptoms of cancer. We believe that CDDP liposomes will help patients with cancer achieve complete remission while improving their QOL.
